# Combination of Gas Plasma and Radiotherapy Has Immunostimulatory Potential and Additive Toxicity in Murine Melanoma Cells In Vitro

**DOI:** 10.3390/ijms21041379

**Published:** 2020-02-18

**Authors:** Gabriella Pasqual-Melo, Sanjeev Kumar Sagwal, Eric Freund, Rajesh Kumar Gandhirajan, Benjamin Frey, Thomas von Woedtke, Udo Gaipl, Sander Bekeschus

**Affiliations:** 1 ZIK Plasmatis, Leibniz Institute for Plasma Science and Technology, Felix-Hausdorff-Straße 2, 17489 Greifswald, Germany; gabriella.pasqual-melo@inp-greifswald.de (G.P.-M.); sanjeev.sagwal@inp-greifswald.de (S.K.S.); eric.freund@inp-greifswald.de (E.F.); rajesh.gandhirajan@inp-greifswald.de (R.K.G.); woedtke@inp-greifswald.de (T.v.W.); 2 Department of General, Visceral, Thoracic and Vascular Surgery, Greifswald University Medical Center, Ferdinand-Sauerbruch-Straße, 17475 Greifswald, Germany; 3 Department of Radiation Oncology, Universitätsklinikum Erlangen, Maximiliansplatz 2, 910524 Erlangen, Germany; Benjamin.Frey@uk-erlangen.de (B.F.); Udo.Gaipl@uk-erlangen.de (U.G.); 4 Institute for Hygiene and Environmental Medicine, Greifswald University Medical Center, Felix-Hausdorff-Straße 2, 17489 Greifswald, Germany

**Keywords:** kINPen, oncology, plasma medicine, reactive oxygen species, ROS

## Abstract

Despite continuous advances in therapy, malignant melanoma is still among the deadliest types of cancer. At the same time, owing to its high plasticity and immunogenicity, melanoma is regarded as a model tumor entity when testing new treatment approaches. Cold physical plasma is a novel anticancer tool that utilizes a plethora of reactive oxygen species (ROS) being deposited on the target cells and tissues. To test whether plasma treatment would enhance the toxicity of an established antitumor therapy, ionizing radiation, we combined both physical treatment modalities targeting B16F10 murine melanoma cell in vitro. Repeated rather than single radiotherapy, in combination with gas plasma-introduced ROS, induced apoptosis and cell cycle arrest in an additive fashion. In tendency, gas plasma treatment sensitized the cells to subsequent radiotherapy rather than the other way around. This was concomitant with increased levels of TNFα, IL6, and GM-CSF in supernatants. Murine JAWS dendritic cells cultured in these supernatants showed an increased expression of cell surface activation markers, such as MHCII and CD83. For PD-L1 and PD-L2, increased expression was observed. Our results are the first to suggest an additive therapeutic effect of gas plasma and radiotherapy, and translational tumor models are needed to develop this concept further.

## 1. Introduction

Melanoma represents the most aggressive and deadliest form of skin cancer [1]. Despite advances in the therapeutic options, patients with advanced melanoma generally have poor outcomes. The 5-year survival rate is about 98% for localized tumor diagnosis but decreases to 62% in patients with regional lymph nodes being affected and to only 17% for widespread metastatic melanoma [2]. To fight against melanoma growth, combination therapies have proven to be most effective because they impact cancer cell biology at multiple signal transduction pathways, resulting in an additive or synergetic effect [3]. 

Radiation therapy has long played a role in the management of all types and stages of melanoma, being used to treat unresectable malignant melanoma or to treat metastatic disease in a palliative setting [4]. Even though melanoma has traditionally been considered as a relatively radio-resistant tumor [5], studies have shown that hypo-fractionated radiotherapy, as well as in combination with immune checkpoint inhibitors [6] or hyperthermia [7], can effectively kill melanoma cells and induce an anti-tumor immune response. With medical gas plasma therapy, another physical treatment modality is emerging in the field of oncology [8].

Cold physical plasma is a partially ionized gas that consists of multiple components such as electrical fields, ions and electrons, thermal and UV radiation, reactive oxygen species (ROS), and visible light [9]. With gas plasma jets, a technology where a noble gas is energized to deliver its chemical reactivity to oxygen and nitrogen in ambient air [10], ROS have been singled out as the main active component achieving the desired biomedical effects [11]. The half-life of many types of ROS is short, and the current concept is that it is not necessarily the plasma-derived ROS that are directly interacting with cells and tissues but rather secondary molecules derived from primary ROS as well as oxidative modifications on organic molecules [12]. When applied at higher doses, these ROS overcome the capacity of tumor cells and activate different cell signaling pathways that culminate in cell death [13,14]. Besides its cytotoxic effect, cold plasma has been described as a potent inducer of the immunogenic cancer cell death (ICD) [15,16]. Here we investigate a new approach to treat malignant melanoma. The approach combines the established radiotherapy with the promising medical gas plasma technology, inducing additive toxicity in murine B16F10 melanoma cells as well as immunomodulatory effects. 

## 2. Results

### 2.1. ROS Generation and Additive Toxicity with Gas Plasma and Single-Dose Radiotherapy

**Figure 1:**
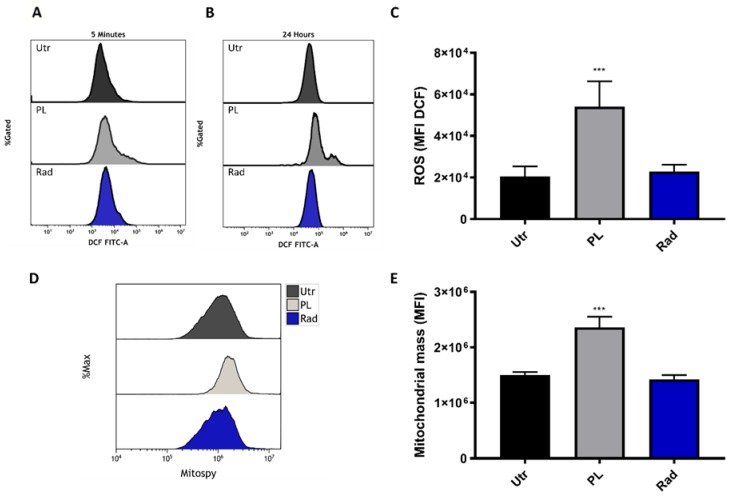
Gas plasma and radiotherapy introduced reactive oxygen and nitrogen species (ROS) in B16F10 melanoma cells. (**A**–**C**) B16F10 cells were exposed either to gas plasma (15 s) or radiotherapy (8 Gy), and intracellular ROS were measured immediately (**A**) and 24 h (**B**) after exposure by flow cytometry, and quantified (**C**); (**D**,**E**) mitochondrial mass was determined by flow cytometry (**D**) and quantified (**E**) at 24 h post-exposure. Data are representative and mean +SE of three experiments. Statistical analysis was performed by one-way ANOVA. Utr = untreated, PL = plasma, Rad = radiotherapy, MFI = mean fluorescent intensity; *** = *p* < 0.001.

**Figure 2:**
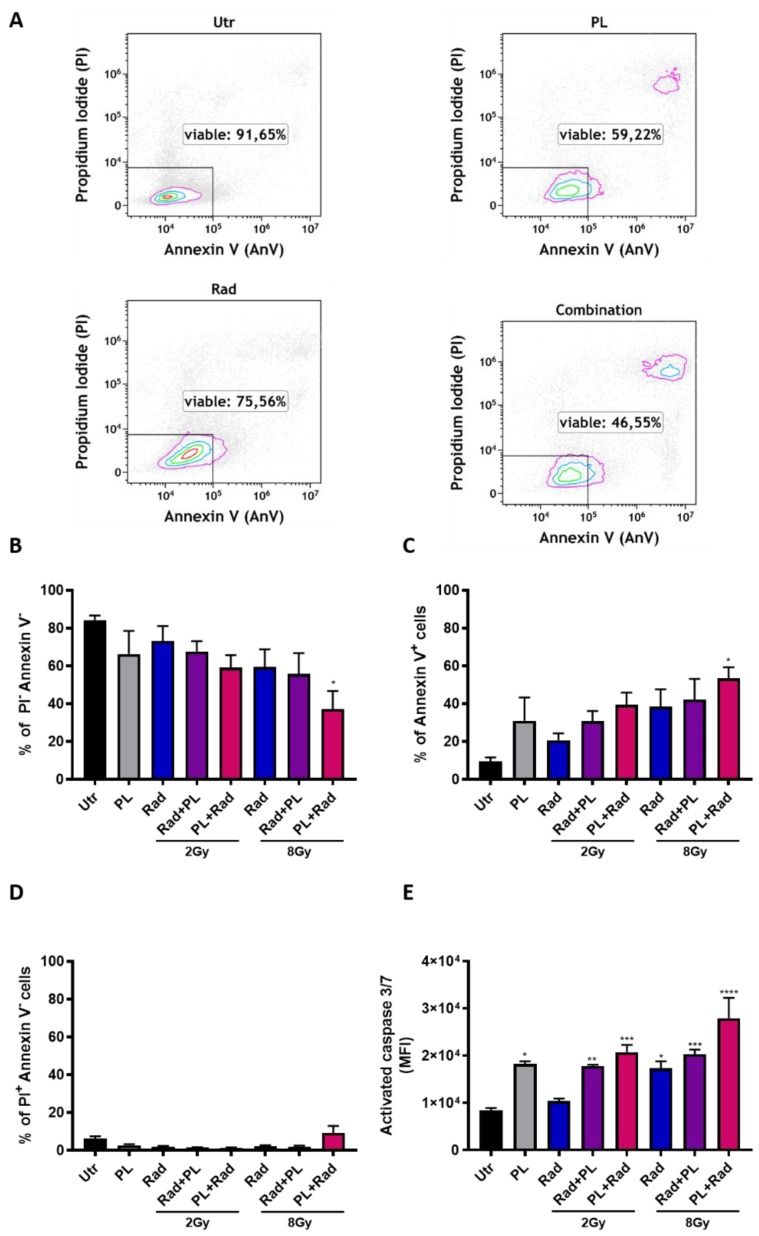
Gas plasma and single-dose radiotherapy induced apoptosis in an additive fashion. (**A**) representative dot plots of Annexin V and propidium iodide (PI) flow cytometric analysis in B16F10 cells 24 h post-exposure to plasma (15 s), radiotherapy, or both; (**B**–**D**) quantification of viable (**B**), apoptotic (**C**), and necrotic (**D**) cells; (**E**) additional confirmation of apoptosis using the cell event caspase 3/7 dye. Data are representative and mean + SE of three experiments. Statistical analysis was performed by one-way ANOVA. Gy = gray, Utr = untreated, PL = plasma, Rad = radiotherapy, MFI = mean fluorescent intensity; * = *p* < 0.05, ** = *p* < 0.01, *** = *p* < 0.001, **** = *p* < 0.0001.

It is well known that cold plasma, as well as ionizing radiation, generates ROS as one mechanism of action. To re-instate this finding with our treatment setup, B16F10 melanoma cells were stained with the redox-sensitive fluorescent dye 2ʹ,7ʹ-dichlorofluorescin diacetate (DCFH-DA) after plasma (15 s) or radiation (8 Gy) treatment and analyzed by flow cytometry. Increased fluorescence intensity was observed after the treatment (Figure 1A) and 24 h later (Figure 1B), which was significant for gas plasma exposure (Figure 1C). Mitochondria is an important endogenous source of ROS. To investigate the mitochondrial mass per cell, B16F10 cells were stained with MitoSpy, a dye that locates in the mitochondrial membrane. Twenty-four hours after treatment, an increased fluorescence was observed in the treatment groups (Figure 1D), which was significant for plasma exposure (Figure 1E). To analyze if cold plasma and ionizing radiation induce additive toxicity in melanoma, B16F10 cells were treated in different combinations of settings and analyzed for apoptosis by flow cytometry (Figure 2A). The combination of cold plasma followed by radiation 8 Gy significantly reduced the number of viable cells (Figure 2B) and increased apoptotic cells (Figure 2C). The number of necrotic cells was inexpressive (Figure 2D). Apoptosis was confirmed via the assessment of the activity of the executioner caspases 3 and 7 (Figure 2E) and was similar in trend as compared to the analysis of phosphatidylserine exposure (Figure 2D). In general, additive toxicity was observed with the combination over the mono treatments, which was more pronounced for the 8 Gy radiation and if plasma was applied prior to radiotherapy.

### 2.2. Plasma and Fractionated Radiotherapy Increased Cell Death and p53 Expression

**Figure 3:**
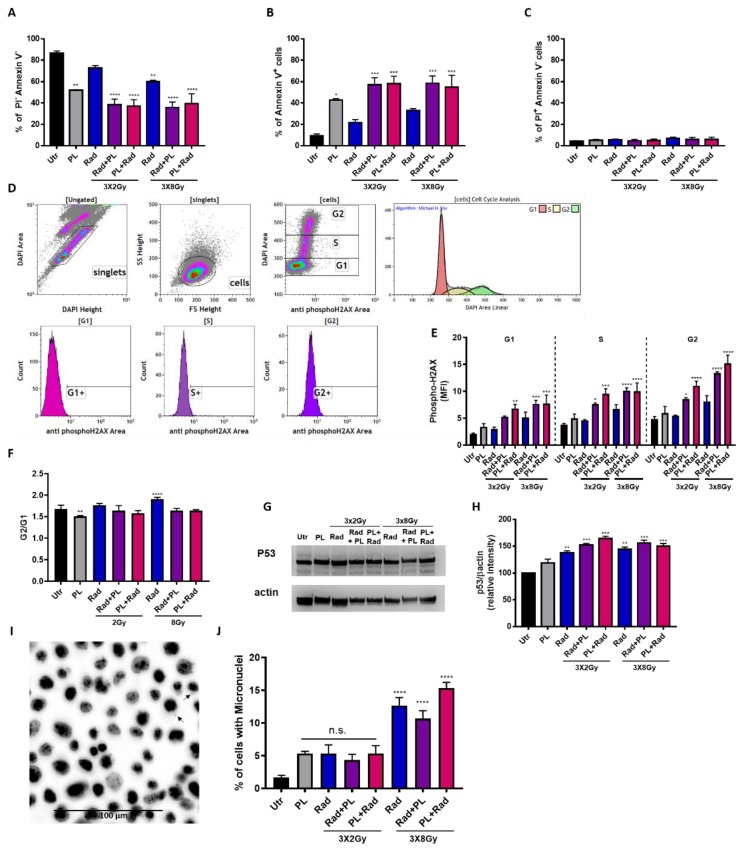
Gas plasma and fractionated radiotherapy conferred toxicity, cell cycle arrest, and p53 upregulation. (**A**–**C**) B16F10 were exposed to plasma (10 s at 0 h), fractionated radiotherapy (0, 24, and 48 h), or both, and viable (**A**), apoptotic (**B**), and necrotic (**C**) cells were analyzed 24 h after the last radiotherapy cycle (total of 72 h post plasma treatment) by flow cytometry; (**D**) gating strategy to analyze cell cycle arrest and cell cycle-dependent H2AX phosphorylation by first gating singlets and the cells to discriminate G1, S, and G2 cell cycle phase as well as algorithm-driven quantification of the phases; (**E**,**F**) quantification of phosphorylated H2AX (**E**) and the G2/G1 ratio (**F**) in B16F10 cells; (**G**,**H**) p53 and actin Western blots (**G**) and protein quantification against β-actin (**H**) in B16F10 cells; (**I**,**J**) representative microscopy image (**I**) and determining the percentage of micronuclei in three fields of view (**J**). Data are representative and mean + SE of three (**A**–**H**) and one (**I**,**J**) experiment. Statistical analysis was performed by one-way ANOVA. Gy = gray, Utr = untreated, PL = plasma, Rad = radiotherapy, MFI = mean fluorescent intensity, n.s. = not significant; * = *p* < 0.05, ** = *p* < 0.01, *** = *p* < 0.001, **** = *p* < 0.0001.

Fractionated radiation settings have been proposed to be more clinically relevant, and we combined this strategy with cold physical plasma treatment. By investigating the amount of viable (Figure 3A) and apoptotic (Figure 3B) cells, a significant additive effect of plasma and radiotherapy was observed, while the number of necrotic cells was unchanged (Figure 3C). Interestingly, the combination with 2 Gy fractionated radiation performed similarly to the 8 Gy fractionated radiation. Notwithstanding, an increase in melanoma cell death was observed in the combination of plasma with both radiotherapy regimens. To elucidate the mechanisms of these findings, cell cycle progression and phosphorylation of the DNA-damage marker histone 2AX (H2AX) was performed simultaneously using flow cytometry (Figure 3D). For γH2AX, the mono treatments gave an increased signal, while the combination enhanced the effects of the mono treatments in an additive fashion and throughout the G1, S, and G2 cell cycle phases (Figure 2E). Again, applying plasma before radiotherapy showed an increased effect size as compared to the opposite sequence being radiotherapy prior to plasma treatment. By contrast, and given the technical limitation of cell cycle analysis, the G2/G1 ratio did not markedly change, suggesting that noticeable changes in the cell cycle might not have occurred (Figure 3F). To understand the involvement of tumor suppressor protein p53, the guardian of the genome, Western blots were performed (Figure 3G), and p53 expression increased in the combination treatment over the mono treatments (Figure 3H). Notably, the 2 Gy fractionated radiation performed similarly to the 8 Gy fractionated radiation. It needs to be mentioned that untreated control cells had unusually high levels of p53, which might have been a result of excess protein loading in the blots. Yet, the images were void of signal saturation, giving accurate results at least in technical aspects. Ionizing radiation is known to induce DNA double-strand breaks (DSBs) as we and others have shown before [17,18]. To this end, we analyzed the number of micronuclei in the mono and combination treatments microscopically (Figure 3I). Micronuclei frequency did not change with mono and 2 Gy combination treatments, while it was significantly elevated with the 8 Gy radiotherapy regimens (Figure 3J). Altogether, fractionated radiotherapy in combination with plasma treatment induced stronger cytotoxicity in melanoma cells. Moreover, an increase in γH2AX formation and p53 expression was observed, and micronuclei formation was found for the 8 Gy radiation treatments.

### 2.3. Plasma and Radiotherapy of B16 Cells and the Immunomodulatory Consequences 

**Figure 4:**
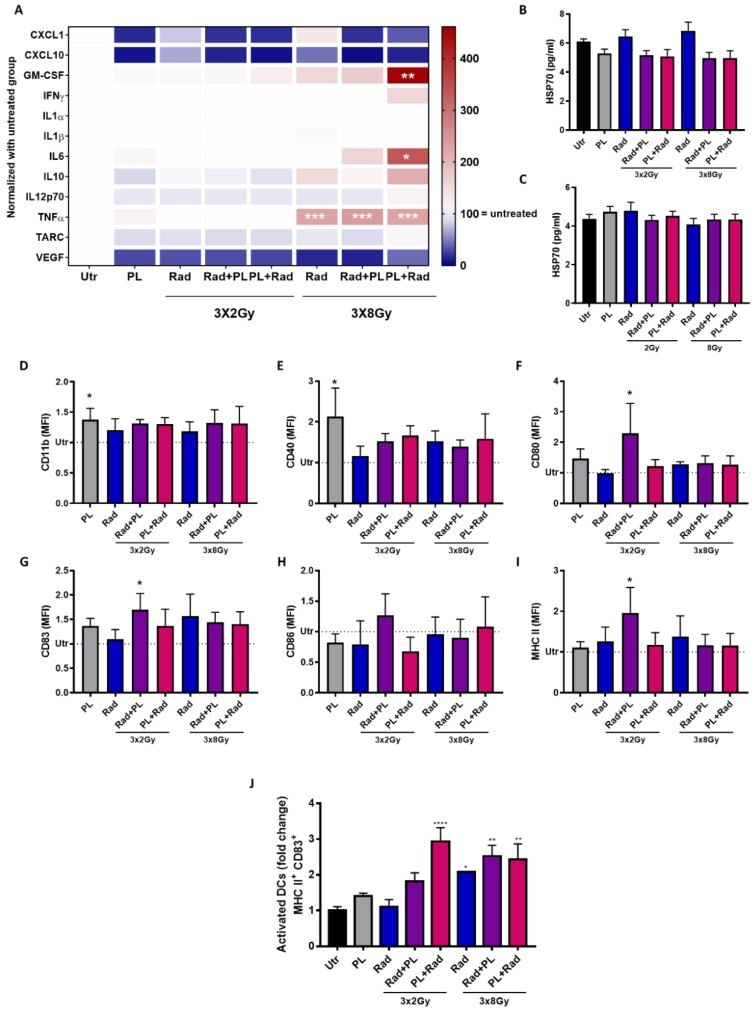
Secretory profile of B16F10 melanoma cells and immunological consequences in dendritic cells. (**A**–**C**) Multiplex cytokine analysis (**A**) and HSP70 ELISA of supernatants of murine B16F10 cells receiving plasma (10 s at 0 h), fractionated radiotherapy (at 0, 24, and 48 h), or both, collected at 24 h after the last radiation dose (**B**), or mono or combination single radiotherapy with plasma (**C**); (**D**–**J**) JAWS murine dendritic cells cultured for 96 h with supernatants of B16F10 cells, and multicolor flow cytometry mean fluorescent intensity (MFI) analysis of activation markers CD11b (**D**), CD40 (**E**), CD80 (**F**), CD83 (**G**), CD86 (**H**), and MHC class II on viable cells (**I**) as well as percentage of MHC class II and CD83 double positive cells (**J**). Data are representative and mean + SE of three experiments. Statistical analysis was performed by one-way ANOVA. Gy = gray, Utr = untreated, PL = plasma, Rad = radiotherapy; * = *p* < 0.05, ** = *p* < 0.01, *** = *p* < 0.001, **** = *p* < 0.0001.

**Figure 5:**
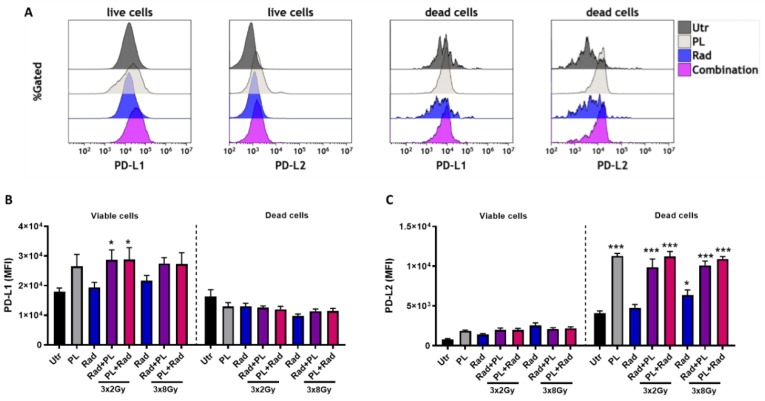
Immune checkpoint analysis in B16F10 melanoma cells. (**A**) representative overlay histograms of PD-L1 and PD-L2 on viable (live) and dead murine B16F10 cells receiving plasma (10 s at 0 h), fractionated radiotherapy (at 0, 24, and 48 h), or both, analyzed by flow cytometry at 24 h after the last radiation dose; (**B**,**C**), quantification of PD-L1 (**B**) and PD-L2 expression. Data are representative and mean + SE of three experiments. Statistical analysis was performed by one-way ANOVA. Gy = gray, Utr = untreated, PL = plasma, Rad = radiotherapy, MFI = mean fluorescent intensity; * = *p* < 0.05, *** = *p* < 0.001.

Evidence showed that both cold plasma and ionizing radiation are able to induce ICD. To this end, the secretory products of tumor cells are vital in immunomodulation [19]. Analyzing the concentration of 12 different cytokines and chemokines in our treatment regimens, no change was observed for CXCL1, CXCL10, IL12p70, and TARC throughout most treatment conditions (Figure 4A). For the 8 Gy treatment regimens, which in general induced stronger changes, a significant increase in GM-CSF, IL6, and TNFα was found when radiotherapy was preceded by gas plasma treatment. The levels of the immunosuppressant IL10 was not significantly changed in all conditions. A prominent damage-associated molecular pattern (DAMP) is HSP70 [20]. Quantification of HSP70 in cell culture supernatants, however, did not give any significant changes with single-dose (Figure 4B) and fractionated (Figure 4C) treatment regimens. To analyze the immunomodulatory consequences of the secretory products of B16F10 melanoma cells exposed to plasma and fractionated radiotherapy in a mono or combination setting, JAWS murine immature dendritic cells (DCs) were cultured with tumor cell supernatants, and their maturation status was investigated using activation surface marker expression multicolor flow cytometry analysis. For CD11b (Figure 4D) and CD40 (Figure 4E), only plasma treatment gave a significant increase when analyzing the mean fluorescent intensities. With CD80 (Figure 4F), CD83 (Figure 4G), CD86 (Figure 4H), and MHCII (Figure 4I), however, the 2 Gy fractionated therapy followed by plasma treatment was significantly increased, except for CD86. The other conditions showed no significant change. When analyzing the percentage of CD83^+^/MHCII^+^ double-positive cells of DCs, a significant elevation was observed with the 8 Gy regimens and the 2 Gy combination that followed plasma treatment (Figure 4J). Hence, we were able to show a prominent modulation of the secretory profile along with an increase in DC maturation with gas plasma and radiotherapy combination treatment. Such engagement is thought to spur antitumor immunity in vivo that strikes on tumors by T-cell activity. However, T-cell activation can be blocked by PD-L1 and PD-L2, and tumors frequently exploit this mechanism to escape immunity [21]. Analyzing both markers on live and dead tumor cells (Figure 5A), we found an increased expression of PD-L1 on live but not dead tumor cells (Figure 5B), and vice versa for PD-L2 (Figure 5C). It may be less common to analyze PD-L1/2 expression on dead cells. However, it is thinkable that, e.g., a lymphocyte contacting a viable tumor cell via T-cell receptor (TCR) major histocompatibility class (MHC) class I interaction may at the same time receive suppressive signals by PDL1/2 engagement of the PD-1 receptor by an adjacent dead tumor cell.

## 3. Discussion

Radiotherapy uses ionizing radiation to target and kill tumor tissue, and it is one of the most standard cancer therapies, being used in more than 50% of patients with cancer [22]. Medical gas plasma therapy is an emerging technology with documented relevance in dermatology [23] and promising significance in oncology [24,25]. Using B16F10 melanoma cells as a model, our aim is to combine both physical modalities to assess its potential in oncology.

In the clinical settings, ionizing radiation can be applied in a single (usually higher) dose, or in a fractionated regimen with several (usually lower) doses. The choice of fractionating comes from the necessity of enabling normal tissue to repair during and after the course of radiotherapy, based on the fact that tumor cells are at a disadvantage since its repair machinery is generally damaged [26]. Furthermore, fractionating the radiation exploits weaknesses in cell cycle processes and DNA damage repair mechanisms of tumor cells, thus resulting in increased tumor cell death induction and possibly local tumor control [27]. In our study, the monotherapy with a single dose of ionizing radiation or plasma gave substantial antitumor toxicity. However, when pretreating the cells with cold plasma, cell death was even more pronounced. Melanoma cells have low rates of spontaneous apoptosis when compared with other tumor types, besides being relatively resistant to apoptosis after chemotherapy [28]. In our study, the combination of plasma and 8 Gy radiation was able to induce caspase 3/7 activation and apoptotic cell death. This additive effect was even stronger with repeated radiotherapy. Interestingly, 3 × 2 Gy was often equally effective than 3 × 8 Gy when preceded by plasma treatment, suggesting that fractionation with low doses might be therapeutically optimal as this regimen gives fewer side effects [29]. The clinical significance of this result is low, however, as cells cultured in 2D have different sensitivity towards radiotherapy as compared to cells in tissues. However, the result serves as a starting point to show the proof-of-concept of combining two different physical approaches. 

Although cancer cells are known to use glycolysis as the primary adenosine triphosphate (ATP) source, melanoma cells rely on mitochondrial oxidative phosphorylation [30]. Plasma exposure may induce a persistent injury to mitochondrial function that caused the mitochondrial mass increase to maintain adequate levels of ATP from oxidative phosphorylation. Besides being a compensatory response, previous authors have suggested that increases in mitochondrial mass could be induced by increased steady-state levels of ROS caused by defective mitochondrial metabolism [31]. Persistent levels of ROS 24 h post-plasma treatment were also found in our study when intracellular ROS interacts with cellular components activating different signaling pathways, causing an initial response [32]. The theory that plasma induces apoptotic cell death through mitochondrial damage by delivering ROS has been proposed by others and us before [33,34]. It has even been proposed that plasma and ionizing radiation share a partly similar behavior for the generation of ROS, especially in the liquid phase of a treated tissue or cell system model [35]. Especially with ionizing radiation, hydroxyl radicals are being generated directly in the nucleus to affect cellular DNA [36].

Double strand breaks (DSBs) represent the most significant DNA damage. Phosphorylated histone H2AX (γH2AX) represents a signal molecule in pathway activation DSBs repair [37]. It is well accepted that DSBs are the primary lesions responsible for the biological effects of ionizing radiation [38]. In our study, the combination with cold plasma and ionizing radiation increased γH2AX throughout all cell cycle phases. However, we recently reported that H2AX phosphorylation in response to plasma treatment is a consequence of pro-apoptotic signaling rather than a direct transfer of gas phase-generated ROS passing through several cellular membranes and compartments to nuclear DNA [39]. The response to DNA damage results in either cell cycle arrest to allow the lesions to be repaired or apoptosis, and p53 is essential in both pathways and can be regulated on both the post-transcriptional and expression levels [40]. Specifically, in cell cycle arrest at the G1 phase, p53 enhances p21 transcription, which, in turn, inhibits cyclin-dependent kinase (CDK) activity [41]. In our study, the combination settings increased p53 expression, which was possibly intertwined with increased γH2AX. Additionally, radiotherapy-induced DNA damage and subsequent misrepair may lead to the formation of complex chromosomal aberrations and micronuclei (MN) that contribute to cell death [42]. For the single treatment regimens (low 3 × 2 Gy fractionated radiotherapy; plasma treatment), a significant change in the number of micronuclei was not observed. This is in line with previous observations in both the radiotherapy [18,43] and plasma medicine field [17,44,45].

A successful cancer therapy includes not only the ability to selectively kill cancer cells but also increases immunogenicity and promotes anti-tumor immunity [46]. For a long time, radiotherapy was known to be a localized form of treatment, with the aim of stopping tumor growth and induction of cell death while the adjacent tissue is minimally affected. Nonetheless, many studies have suggested ionizing radiation also triggers an anti-cancer immune response [47]. In ICD, cells release DAMPs such as HSP70 in a spatiotemporal manner that act as danger signals and activate antigen-presenting cells [48]. In our study, we did not observe a release of pro-immunogenic molecules such as HSP70, CXCL1, and CXCL10. By contrast, such effects and ICD were shown before for both radiotherapy [49,50,51] and gas plasma treatment in tumor and melanoma cells [52,53,54]. The difference might have been due to different sampling time points and treatment regimens. The secretory profile, however, has shown a pronounced increase of GM-CSF, IL6, and TNFα, albeit only in the 3 × 8 Gy radiation regimens. The molecules have been implicated in anti-melanoma immunity. For instance, GM-CSF was included in anti-melanoma vaccination efforts [55], and TNFα was previous a part of anti-melanoma clinical strategies [56]. Depending on the treatment regimen, IL10 levels were either increased or decreased in our study. Although being considered a classical pro-tumor cytokine [57], new evidence has emerged showing a potential immuno-stimulating activity of IL-10 that may support CD8^+^ T-cell activity [58,59,60]. The immunomodulatory action of combined plasma and radiotherapy in B16 cells was further supported by an increase of maturation markers in DCs in our study. GM-CSF is known to promote dendritic cell activation, which is concomitant with an upregulation of MHCII and CD83 in these cells [61]. Nevertheless, we also observed an increased expression of PD-L1 on live melanoma cells. This ligand assists in the immune escape of tumors, negatively regulating T-cell activity. Its up-regulated expression had already been described as an unwanted effect of ionizing radiation [62]. In order to counteract immunosuppressive effects and exploit the immune-activating properties of the studied combination, immune checkpoint inhibitors can be used [62,63,64,65]. The approved antibodies anti-PD-L1 nivolumab and pembrolizumab were shown to have positive effects in melanoma treatment [66].

## 4. Materials and Methods

### 4.1. Cell Culture and Treatment

B16F10 cells (ATCC CRL-6475) were cultured in Roswell Park Memorial Institute (RPMI) medium 1640 supplemented with 10% fetal bovine serum (FCS) and 100 U/mL penicillin and 0.1 mg/mL streptomycin (all Gibco, Dublin, Ireland). For the experiments with a single radiation dosage, 7.5 × 10^4^ cells in 1 mL of fully supplemented cell culture medium were placed in wells of a 24-well plate. Cell suspensions were treated with the atmospheric pressure argon plasma jet kINPen using two standard liters per minute (Air Liquid, Paris, France). The treatment time was 15 s, followed by ionizing radiation (GE Inspection Technologies, Hürth, Germany) at a setting (120 kV, 22.7 mA, 0.5 min) delivering either 2 or 8 Gy. The sequence of treatment was also swapped, with first ionizing radiation followed by gas plasma treatment. Cells were incubated for 24 h, and supernatants and cells were harvested for downstream analysis. For the experiments with fractionated radiotherapy, B16F10 cells in 500 µL of fully supplemented culture medium were added to 24-well plates at different densities, being 2.5 × 10^4^ for untreated control and 2 Gy radiation treatment groups, and 7.5 × 10^4^ for the other groups. Gas plasma treatment was performed for 10 s, followed by ionizing radiation (2 or 8 Gy), or vice versa. After 24 and 48 h, the radiation groups received a second and third dose of ionizing radiation, respectively. After another 24 h of incubation after the last radiation dose (totaling to 72 h after the first plasma or radiotherapy), cells and supernatants were collected for downstream analysis.

### 4.2. Flow Cytometry and Micronuclei Analysis

ROS levels were assessed using 2′,7′-dichlorodihydrofluorescein diacetate (DCFH-DA; ThermoFisher, Waltham, MA, USA), which was converted to highly fluorescent 2′,7′-dichlorofluorescein (DCF) in the presence of ROS. B16F10 cells were treated with cold plasma (15 s) or ionizing radiation (8 Gy) and incubated with 5 µM of DCFH-DA (37 °C, 30 min) immediately after or 24 h after the treatment. Analysis of the mean fluorescence intensity (MFI) of DCF in viable cells was subsequently performed using a flow cytometer (CytoFLEX S; Beckman-Coulter, Brea, CA, USA). Total mitochondrial mass was analyzed at the same conditions using MitoSpy (37 °C, 15 min; BioLegend, London, UK) via flow cytometry. Cell viability was evaluated 24 h after the last treatment with both single and fractionated therapy. After cells were detached with accutase, they were washed and resuspended in annexin V binding buffer and stained with annexin V fluorescein isothiocyanate (FITC) and propidium iodide (PI) at 4 °C for 30 min (all BioLegend, London, UK). To quantify the activation of executioner caspases within cells, staining of cell suspensions was done using the CellEvent caspase 3/7 dye (life technologies, Carlsbad, CA, USA) prior to analysis using flow cytometry. To quantify the expression of programmed cell death ligand (PD-L) 1 and 2, cell suspensions were incubated with monoclonal antibodies targeting PD-L1 conjugated to phycoerythrin-cyanine 7 (PE-Cy; eBioscience, San Diego, CA, USA) and PD-L2 conjugated to allophycocyanin (APC; BioLegend, London, UK). Zombie dye Aqua (BioLegend, London, UK) was used to discriminate live from dead cells. The acquisition was performed using flow cytometry. For analysis of cell cycle and nuclear phosphorylation of histone 2AX (γH2AX), cells were collected, fixed in 70% ethanol (−20 °C), and washed. Staining was performed with 10 µM of 4′,6-diamidino-2-phenylindol (DAPI) and anti-γH2AX monoclonal antibodies conjugated with Alexa Fluor (AF) 647 (all BioLegend, London, UK) for 20 min. After washing, cells were analyzed by flow cytometry (Gallios; Beckman-Coulter, Brea, CA, USA). Another set of the fixed and DAPI stained cells was added to the wells of a 96-well plate and imaged with a 20× objective (NA 0.4; Zeiss, Jena, Germany) using a high throughput imaging system (Operetta CLS; Perkin Elmer; Hamburg, Germany). The quantification of micronuclei was performed manually. 

### 4.3. Immunoblotting

Following the indicated incubation, cells were harvested in ice-cold PBS and lysed in RIPA buffer (ThermoFisher, Waltham, MA, USA) supplemented with complete protease and phosphatase inhibitors (PIM complete; Roche, Basal, Switzerland) for 20 min on ice. After centrifugation at 15,000× *g* for 15 min at 4 °C, the total protein in the whole-cell extracts was quantified using Rotiquant (Carl Roth, Karlsruhe, Germany). Twenty-five micrograms of protein were resolved by SDS-PAGE and blotted on polyvinylidene fluoride membranes (both Invitrogen, Carlsbad, CA, USA). The membranes were probed with primary anti-p53 and anti-β-actin antibodies, followed by anti-rabbit antibodies coupled to horseradish peroxidase (all Santa Cruz, Dallas, TX, USA). Signals were acquired in a chemiluminescence detection system (Applied Biosystems, Foster City, CA, USA) in a linear dynamic range mode. 

### 4.4. Analysis of Cell Culture Supernatants

Enzyme-labeled immunosorbent assay (ELISA) was performed to quantify heat-shock protein (HSP) 70 (R&D Systems, Minneapolis, MN, USA) according to the manufacturer’s instructions. To analyze the concentration of 12 different chemokines and cytokines, a bead-based multiplex assay (LEGENDplex; BioLegend, London, UK) was used to analyze chemokine (C-X-C motif) ligand (CXCL) 1 and 10, granulocyte-macrophage colony-stimulating factor (GM-CSF), interferon (IFN) γ, interleukin (IL) 1α, IL1β, IL6, IL10, IL12p70, tumor necrosis factor (TNF) α, chemokine (C-C motif) ligand 17 (CCL17) (also known as TARC), and vascular endothelial growth factor (VEGF) simultaneously. The assay was performed according to the manufacturer’s instructions, using the principle of sandwich immunoassays. The quantification of the MFI of each bead population capturing a single analyte against a known standard was performed using flow cytometry (CytoFLEX S; Beckman-Coulter, Brea, CA, USA) and dedicated software (VigeneTech, Carlisle, MA, USA).

### 4.5. Dendritic Cell Analysis

Immature JAWS II (JAWS, ATCC CRL-11904) murine dendritic cells (DCs) were cultivated in Iscove’s modified Dulbecco’s media (IMDM) supplemented with 20% FCS Gold Plus (all Bio&Sell, Feucht, Germany), 5 ng/mL mouse GM-CSF (Invitrogen, Carlsbad, CA, USA) and 1% penicillin and streptomycin. For the experiment, 5 × 10^3^ cells in 20 µL of fully supplemented IMDM were plated in wells of 96-well plate. Wells were then supplemented with 80 µL of the respective B16F10 cell culture supernatants. This did not affect the viability of the cells. After 96 h, the cells were harvested with accutase and stained with DAPI and anti-mouse monoclonal antibodies (conjugate) targeting CD11b (AF488), CD40 (PE), CD86 (PE-Cy7), CD83 (APC), I-A/I-E (MHCII; APC-Cy7), and CD80 (brilliant violet (BV) 650) for 30 min on ice. After washing, flow cytometric analysis was performed using flow cytometry (CytoFLEX S; Beckman-Coulter, Brea, CA, USA).

### 4.6. Statistical Analysis

Graphing and statistical analysis were performed using Prism 8.3 (GraphPad Software, San Diego, CA, USA). Mean and standard errors were calculated using the one-way analysis of variances (ANOVA). Flow cytometric data were analyzed using Kaluza 2.1 software (Beckman-Coulter, Brea, CA, USA).

## 5. Conclusions

Gas plasma and radiotherapy showed additive effects in terms of cytotoxicity, cell cycle arrest, and release of immunostimulatory products in murine melanoma cells. Repeated (fractionated) radiotherapy was more efficient than the single-dose regimen. In tendency, combination therapy was more effective when applying the plasma first and the radiotherapy second as compared to the vice versa situation. The translational relevance of these findings needs to be elaborated in future research using syngeneic tumor models to further study the effect of combination therapy on the tumor microenvironment and metastasis.
